# Sexual Dimorphism and Menopausal Transition: A Narrative Review of the Metabolic and Physical Effects of Intermittent Fasting

**DOI:** 10.3390/nu18091344

**Published:** 2026-04-24

**Authors:** Alexsandra Rojas Drinnon, Andres Calderon, Maheswaran Dhanasekaran, Jawairia Shakil, Bhargavi Patham

**Affiliations:** 1 Houston Methodist Academic Institute, Houston, TX 77030, USAmdhanasekaran@houstonmethodist.org (M.D.); jshakil@houstonmethodist.org (J.S.); 2 Piedmont Physicians Endocrinology Covington, Covington, GA 30014, USA; acalderonv.93@gmail.com

**Keywords:** fasting, cardiometabolic, sex, post-menopause, obesity, weight loss

## Abstract

The global rise in obesity and cardiometabolic disease represents a major public health concern and contributes significantly to cardiovascular morbidity and mortality. Contemporary Western dietary patterns and excess adiposity are strongly associated with atherosclerotic cardiovascular disease. Although pharmacologic therapies have expanded, lifestyle interventions remain the cornerstone of prevention and management. However, identifying sustainable and effective dietary approaches continues to be challenging given the wide range of available nutrition regimens. Intermittent fasting (IF) has emerged as a promising strategy for weight reduction and metabolic improvement. In this article, we review the physiological effects of IF, including metabolic switching, ketosis, and improvements in insulin sensitivity and inflammatory regulation. We also evaluate clinical evidence regarding the impact on cardiovascular risk, as well as its safety and tolerability. We examine the hormonal responses to IF based on sex. While early studies raised concerns regarding potential reproductive and endocrine disturbances, recent data suggest beneficial effects in both males and females. IF may modestly reduce testosterone in men without impairing muscle mass or strength and may improve metabolic and reproductive outcomes in women, particularly those with hyperandrogenic conditions such as polycystic ovarian syndrome, with favorable effects also observed in postmenopausal women, especially when combined with physical activity.

## 1. Introduction and Overview

In recent decades, non-communicable diseases have become the leading global causes of morbidity and mortality. Cardiovascular disease, particularly ischemic heart disease (IHD), remains the primary contributor to death worldwide. In the United States, heart disease accounted for 167.2 deaths per 100,000 individuals in 2023 and has remained the leading cause of death for over 70 years [1]. Global data identify similar patterns, with IHD as the top cause of mortality between 1990 and 2023, with atherosclerotic cardiovascular diseases collectively responsible for approximately 25% of all deaths. Importantly, a large proportion of this burden is attributable to modifiable risk factors, including hypertension, elevated non–HDL cholesterol, smoking, diabetes, and abdominal obesity, which together account for more than half of IHD cases in both women and men [2,3]. Western dietary patterns, characterized by high consumption of processed meats, refined grains, sugary beverages, and fried foods, are strongly associated with increased risk of IHD and stroke. In contrast, diets rich in fruits, vegetables, whole grains, and healthy fats are linked to lower cardiovascular risk [4,5,6]. Conversely, adherence to dietary patterns such as the Mediterranean diet has been shown to significantly reduce cardiovascular events, underscoring the importance of nutrition in disease prevention [7].

In comparison to other dietary patterns, intermittent fasting (IF) has gained attention as an effective lifestyle strategy to improve metabolic and cardiovascular health. Cycling between periods of eating and fasting promotes a metabolic switch from glucose to fat utilization, enhancing insulin sensitivity, reducing inflammation, and improving energy regulation. These physiological changes are associated with weight loss, better glycemic control, lower blood pressure, and favorable effects on body composition. Importantly, IF offers a flexible and sustainable approach for many individuals, which may improve long-term adherence compared with continuous calorie restriction. Emerging evidence also suggests potential benefits for hormonal balance, liver health, and cellular repair processes such as autophagy. Given the growing burden of obesity, diabetes, and cardiovascular disease, IF represents a promising, low-cost, and accessible dietary strategy to support overall health when combined with balanced nutrition and regular physical activity. However, significant knowledge gaps limit the clinical application of IF, particularly in women. Optimal protocols across life stages remain unclear, and data on reproductive outcomes and long-term safety, particularly bone health, are insufficient. Mechanisms underlying sex-specific responses are poorly understood, and many studies are underpowered and underrepresent perimenopausal women.

The aim of this article is to provide a comprehensive review of IF as a dietary strategy, examining its physiological, metabolic, and clinical effects across diverse populations. We explore the underlying mechanisms of IF and evaluate its impact on weight management, glycemic control, cardiovascular risk factors, and overall metabolic health, with particular attention to sex-specific responses, including reproductive hormones, fertility, and muscle metabolism in both males and females. Additionally, we examine the effects of IF in premenopausal and postmenopausal women, considering the interplay between hormonal changes, body composition, and cardiometabolic outcomes.

## 2. Methodology

This structured narrative review was conducted to synthesize mechanistic, clinical, and translational evidence on IF across diverse study designs, including randomized controlled trials, systematic reviews and meta-analyses, observational cohort studies, mechanistic human studies, and animal models. A comprehensive literature search was performed in PubMed/MEDLINE for studies published between December 2015 and December 2025, reflecting the period of rapid advancement in this field. The search strategy incorporated both Medical Subject Headings (MeSH) and free-text terms, combined using Boolean operators across predefined thematic domains (detailed in Supplementary Table S1). Eligible studies included those evaluating recognized IF regimens—such as time-restricted eating, alternate-day fasting, modified alternate-day fasting, and the 5:2 approach—in human or animal models, with outcomes related to metabolic, cardiometabolic, or hormonal parameters. Randomized and controlled clinical studies, systematic reviews, narrative reviews with mechanistic insights, and observational studies reporting sex- or menopause-specific outcomes were included. Studies were excluded if they lacked relevant outcomes, focused on pediatric populations, evaluated religious fasting without metabolic data, or were non-English, duplicate, or without accessible full text (Supplementary Table S2).

All retrieved records were imported into a reference management system and de-duplicated, followed by manual screening of reference lists to identify additional relevant studies. Data extraction included study characteristics (author, year, design), population details (sample size, age, sex distribution, menopausal status), IF protocol and duration, and metabolic and hormonal outcomes. (Supplementary Table S3) Due to substantial heterogeneity in study designs, populations, interventions, and outcomes, a narrative synthesis approach was employed. Findings were organized into five thematic domains: physiological mechanisms (including metabolic switching, ketogenesis, and neuroendocrine regulation), cardiometabolic effects (weight, glycemic control, lipid profile, blood pressure, inflammation), safety and tolerability, sex-specific metabolic and hormonal responses, and the impact of IF across menopausal transition and postmenopausal health. Mechanistic and animal studies were used solely to support biological plausibility and were not considered for drawing clinical conclusions.

During the preparation of this manuscript, the author(s) used Open Evidence (version 2) for the purposes of creating organized tables of existing comparison data. The authors have reviewed and edited the output and take full responsibility for the content of this publication.

## 3. Biological and Clinical Effects of Intermittent Fasting

Observations of prolonged fasting done for cultural or religious reasons inspired the introduction of time-restricted eating for cardiometabolic health and have been extensively studied from the biological to clinical perspectives. Results of IF for religious or cultural purposes are reviewed in depth elsewhere [8]. The metabolic switch from glucose to cell-derived ketone energy consumption, shown in animals, explains the changes in energy use during periods of fasting as an adaptive response. Classically, fasting, defined as the cessation of caloric ingestion, in the post-absorptive state, induces glycogenolysis with the phosphorylation of glycogen to release glucose monomers from its macrostructure in the liver for systemic utilization and from the muscle for local utilization. The liver glycogen storage can sustain glucose use between 12 and 24 h, depending on the basal metabolic state; however, even overnight fasting can significantly lower the glycogen storage content [9] and requires a hypo-insulinemic environment. After glycogen storage depletion, energy utilization is sustained by gluconeogenesis for sustained glucose-dependent metabolism. The cycle utilizes lactate and fat- and glycogenic amino acid-derived glycerol as substrate for the de novo conversion of glucose. This cycle has the highest performance during the first 24 to 48 h after the onset of fasting, and its efficiency decreases over the next few days, while energy utilization switches to ketosis. Ketogenesis entails the oxidation of free fatty acids in the hepatic mitochondrion and aims to produce ketone bodies that will enter a metabolic pathway that ends in the production of acetyl CoA in target organs such as the brain. The highest efficiency of ketone bodies production is reached at 48 h after the onset of fasting; however, it starts as early as 8 h. With fasting periods, not only does calorie intake decrease considerably, but ketosis is known to be beneficial from a metabolic perspective. During high ketone body circulation, there is improvement in glycemic control, blood pressure, heart rate, and decreased energy requirements, decreased body mass, as well as improved inflammatory regulation (Table 1 summarizes the clinical benefits of IF) [10,11,12].

Beyond the metabolic switch, fasting plays a role in hormone regulation at the hypothalamic level. Early observations connected the negative energy balance with changes in reproductive physiology via kisspeptin signaling and hypothalamic-pituitary-gonadal axis (HPG). Kisspeptin is a hypothalamic neuropeptide encoded by the *KISS1* gene. It stimulates gonadotropin-releasing hormone (GnRH) neurons through the *KISS1* receptor, promoting pulsatile GnRH secretion and release of luteinizing hormone (LH) and follicle-stimulating hormone (FSH) from the pituitary. During energy deficiency, reduced leptin and insulin suppress kisspeptin activity, leading to decreased GnRH pulsatility and hypogonadotropic hypogonadism [13]. Animal studies have demonstrated that in an acute fasting state, there is a decrease in LH with an increase in FSH in female rats, whereas in male rats, acute fasting did not alter the concentration of these hormones [14]. Furthermore, through a kisspeptin-unrelated mechanism, fasting causes an orexigenic hormonal cascade with elevation of neuropeptide Y, aguti-related peptide (AgRP), and a decrease in insulin concentrations, which cause a direct decrease in GnRH pulses and, in extreme conditions, can cause hypothalamic hypogonadism [15,16].

From a clinical perspective, different IF regimens have been proposed. Alternate-day fasting (ADF) involves an alternating day of no calorie consumption followed by a day of liberalized calorie intake. The modified ADF (MADF) concept is similar to regular ADF for two days but it restricts the calorie intake on non-fasting days for five days in the week. Time-restricted eating (TRE) requires a period of time every day where calorie consumption is liberalized, followed by a longer period of zero calorie intake, each day, usually used with schedules of a 4–10 h feeding window, with 14 to 20 h of fasting.

A network meta-analysis comparing different IF strategies showed that ADF had the highest weight effect, with a mean weight loss of −3.63 kg compared to a liberalized diet, followed by TRE with 2.46 kg mean weight loss, in patients who sustained each diet for more than 24 weeks [7]. In a controlled trial, patients with obesity were enrolled in either TRE plus energy restriction or a self-selected eating window of more than 12 h. The patients assigned to TRE had a weight loss difference of −2.3 kg compared to the control group [11], although there were no major changes in fat mass and waist circumference.

In a randomized controlled trial, IF compared to continuous energy restriction for 12 months, found that the glycemic improvement in patients with diabetes was comparable, achieving a 0.3 and 0.5% reduction in A1c [17]. When compared to metformin and empagliflozin, a 5:2 schedule of IF (MADF) showed the greatest A1c reduction of 1.9%, versus 1.6% and 1.5% reductions, respectively. Cardiovascular benefits of IF derive from improvement in blood pressure, with a mean 4 mmHg decrease in diastolic blood pressure in patients following TRE. Lipid profile has shown a variable response to IF, and no consistent results are available in the published literature. To the extent of the literature review, there is no direct analysis of the incidence of cardiovascular events in patients following intermittent diet strategies compared to the incidence rate in patients following a liberalized diet.

Although considered safe and well-tolerated, the detrimental effects of IF have also been studied. Reports have concluded that most side effects are in the order of increased hunger and irritability, which tend to improve after the first month. A meta-analysis including more than 1300 patients found that fatigue was the most common symptom associated with IF, present in 14% of the patients, headache in 13%, and dizziness in 10%. None of these results were statistically significant when compared to the control groups. Notably, most of the IF-controlled trials had at least more than 80% patient adherence to the studied regimen [18].

Although not intended for cardiometabolic purposes, religious fasting carries the risk of hypoglycemia and dehydration, especially in patients with diabetes with concurrent use of glucose-lowering medications, for which the American Diabetes Association has implemented recommendations to assess the risk and prevention of hypoglycemia in such populations [19].

**Table 1:** Effects of intermittent fasting.

Parameter	Effect	Reference
Diabetes	1.9% A1c reduction	[20]
Weight	9.7 kg reduction	[20]
HOMA-IR	0.60 index reduction	[21]
Lipid profile	6.4 mg/dL reduction in LDL, No statistical change in HLD, total cholesterol and triglyceride concentration.	[21]
Blood pressure	4 mmHg reduction in diastolic blood pressure	[19]
Inflammatory markers	0.024 mg/dL reduction in C-reactive protein 0.16 pg/mL reduction in TNF-⍺ 0.54 pg/mL reduction in IL-6	[22]
Major cardiovascular outcomes	0.9 to 1.7 points reduction in Framigham Risk Scores	[23]

## 4. Intermittent Fasting in Males vs. Females

While previous data have demonstrated noninferiority and, in some studies, superiority of IF compared to continuous caloric restriction, it is important to recognize that the pre-clinical studies using rodents included only male specimens [24]. Thus, the effects of IF in both males and females and its potential gender-based differential effects remain a question.

Multiple studies in recent years, both human and animal, have aimed to determine this. Among the most notable is a 2013 study by Kumar et al. which aimed to determine the effects of IF on the hypothalamo-hypophysial-gonadal axis in both male and female rats [25]. After three months, regardless of sex, both groups demonstrated a reduction in body weight and improved glucose concentration. In addition, reproductive changes, namely disturbed estrous cycles and reduced ovarian weight in females and decreased testosterone in males, were noticed. Interestingly, these reproductive alterations were more pronounced in females, raising concern for adverse reproductive side effects in this population [25].

Though this study has sparked conversations about reproductive health in young women attempting IF, it is important to note that the study population comprised younger rat populations, amounting to adolescent or underage women around nine years of age in the human population [26]. Furthermore, emerging evidence highlights additional mediators—such as gonadotropin-inhibitory hormone (GnIH/RFRP-3) and glucocorticoid signaling—that contribute to stress-induced reproductive dysfunction and may not be fully captured by current models.

More recent studies have continued to study the sex-based differences in IF (Table 2 outlines the sex-specific metabolic and physical effects of intermittent fasting). In 2024, Bosch et al. followed rats first during a fed phase, in which rat participants were exposed to either “cafeteria” food similar to the typical Western diet or standard rat chow (the former of which caused up to a 101% weight gain in females and 44% weight gain in males) [24]. Following this, both categories of rats were then subjected to either a diet based on caloric restriction or IF [24]. Notably, both sexes, regardless of initial diet, in the time-restricted eating groups did not develop any further fatty liver accrual and demonstrated improved glucose regulation. However, only the female subjects demonstrated comparable weight loss in both the caloric restriction and IF groups. Contrary to this, males demonstrated significantly more weight loss when undergoing caloric restriction compared to IF [24]. Given these findings, if translated to human females, IF offers an alternative to traditional caloric restriction without sacrificing weight loss.

Furthermore, multiple studies have investigated sex-based differences in muscle gain while intermittent fasting compared to caloric restriction. Overall, when combining either resistance training or high-intensity interval training with IF in females, muscle hypertrophy and gains were not decreased compared to caloric restriction [27,28]. In males, data are similar, demonstrating increased upper and lower body muscular endurance in the time-restricted fasting group compared to control [29] or, at a minimum, muscle area and strength between both time-restricted eating groups and normal diet or caloric restriction groups remained unchanged [29].

Hormonally, while it is true that recent studies have demonstrated IF-induced hormonal changes in both males and females, these changes likely cause little to no harm and may even be beneficial to some populations. In premenopausal, obese women, IF has been shown to decrease androgen concentration [26]. In addition, males also demonstrate decreased testosterone without a significant impact on muscle mass or muscular strength [26]. However, it is recognized that reduced testosterone concentration in males could result in decreased libido. In contrast, females with underlying hyperandrogenism, such as polycystic ovarian syndrome, could experience improved menstruation and fertility [26]. In fact, 5 weeks of time-restricted eating has shown improved endocrine and metabolic profiles in women with anovulatory polycystic ovarian syndrome [30]. This included more regular menstruation, improved gonadal hormone profiles, decreased BMI, and improved insulin resistance without much effect on LH or FSH [30]. Seemingly, these data contradict previous concerns of deleterious reproductive effects in young females and instead may indicate an overall benefit to certain populations.

Overall, while both males and females demonstrate improved metabolic profiles following initiation of IF, the differential hormonal effects should be taken into consideration.

**Table 2:** Metabolic and physical changes in intermittent fasting in adult males vs. adult females.

Parameter	Adult Males	Adult Females	References
Body Weight Change	−6.2 ± 4.4% (12-week ADF) −2.5% (22-day ADF, healthy lean males) −8% (6-month IF, middle-aged with overweight)	−4.6 ± 3.2% (premenopausal, 12-week ADF) −6.5 ± 3.2% (postmenopausal, 12-week ADF) −8% (6-month IF, middle-aged with overweight)	[21,26]
Lean Mass	Decreased similarly to females (main effect of time, *p* < 0.05) Muscle mass and strength not negatively affected despite testosterone reduction	Decreased similarly to males (main effect of time, *p* < 0.05)	[31]
Fasting Insulin	−57% decrease (22-day ADF, healthy lean males) Decreased similarly to females (main effect of time, *p* < 0.05)	Decreased similarly to males (main effect of time, *p* < 0.05) Greater increase in insulin sensitivity with 5:2 IF vs. continuous caloric restriction	[26]
Fasting Glucose	Significant reduction (WMD = −3.34 mg/dL, *p* = 0.024)	Significant reduction (WMD = −3.34 mg/dL, *p* = 0.024)	[12]
HbA1c	Significant reduction (WMD = −0.08%, *p* = 0.005)	Significant reduction (WMD = −0.08%, *p* = 0.005)	[21]
Total Cholesterol	Lowered with IF	Lowered with IF	[10]
TNF-α	Significant reduction (SMD = −0.31, *p* = 0.009) Time-restricted feeding showed largest reduction (−0.39, *p* = 0.001)	Significant reduction (SMD = −0.31, *p* = 0.009)	[32]
C-Reactive Protein (CRP)	Significant reduction (SMD = −0.19, *p* = 0.04) 5:2 diet ranked highest for CRP reduction	Significant reduction (SMD = −0.19, *p* = 0.04)	[32]
Leptin	Significant reduction (SMD = −0.57, *p* = 0.005)	Significant reduction (SMD = −0.57, *p* = 0.005)	[32]
Testosterone	Reduced in lean, physically active young males with IF	Decreased with IF in premenopausal women with obesity	[26]
Sex Hormone-Binding Globulin (SHBG)	No significant effect with IF	Increased with IF in premenopausal women with obesity	[26]
Estrogen	Not measured in comparative studies	No significant effect with IF	[26]
Adherence to IF Protocol	~6.2 days/week (similar to females) ~400–500 kcal excess consumed on fast days	~6.2 days/week (similar to males) ~400–500 kcal excess consumed on fast days	[31]

## 5. Menopausal Influences of Intermittent Fasting

Given the potential hormonal benefits of IF on reproductive-age females, it begs the question of whether post-menopausal women will achieve similar benefits. Menopause is characterized by physiologic and hormonal changes, including but not limited to decreasing estrogen concentration. These changes often translate to increased visceral and/or subcutaneous fat, decreased muscle mass, and increased risk of obesity, diabetes, hypertension, and hypertriglyceridemia in later years [33,34].

Recent studies have aimed to identify if IF can mitigate these detrimental symptoms of menopause. Like premenopausal women, IF seems to improve insulin sensitivity and promote fat oxidation [34]. IF groups demonstrated overall improvement in physical markers such as waist circumference, waist-to-height ratio, maximal oxygen intake and average heart rate [34,35]. Moreover, IF studies that demonstrated these findings were studied in conjunction with exercise programs such as high-intensity interval training (HIIT) or resistance training. Repeatedly, those who remained sedentary achieved minimal to no benefit, emphasizing the importance of both diet and physical activity.

Post-menopausal women demonstrate similar hormonal and metabolic benefits from IF compared to younger women, including improved glucose and insulin concentrations (Table 3 summarizes the differential effects of intermittent fasting on metabolic, physical, and hormonal markers by menopausal status) [35]. In addition, some studies have also demonstrated a decrease in serum cortisol concentration with IF, potentially improving stress-related hormonal imbalances, and an increase in DHEAS, a hormone that usually declines during menopause and may contribute to menopausal symptoms [33]. Interestingly, there is some indication that time-restricted or intermittent fasting (IF) may enhance activity in the brain’s reward circuitry, particularly the mesolimbic dopamine system, which could potentially boost exercise and dietary motivation and adherence in postmenopausal women [34].

In addition, bone health and muscle preservation are critical concerns for postmenopausal women undertaking caloric or intermittent fasting, as these interventions can reduce bone mineral density and accelerate sarcopenia, particularly without concurrent exercise. Furthermore, micronutrient deficiencies, common in these dietary patterns, further exacerbate risks, especially for calcium, vitamin D, and protein intake. Ensuring adequate nutrition and resistance exercise is therefore essential to protect musculoskeletal health during such interventions.

Overall, similar to premenopausal women, IF in conjunction with exercise has the potential to offer unique benefits for post-menopausal women.

## 6. Conclusions

It is evident that IF has been shown to exert significant effects on metabolic, cardiovascular, and hormonal health, including improvements in weight management, insulin sensitivity, inflammation, and energy regulation. The current evidence suggests that neither male nor female dieters have significant detrimental side effects outside of expected hunger and potentially mild hormonal changes in men. To the contrary, IF may well be beneficial for menstruation and post-menopausal symptoms in females of all ages. Studies generally indicate that IF is safe and well-tolerated, with most side effects being mild and transient, making it easy to follow. Despite the growing body of evidence, several areas warrant further investigation, such as exploring the long-term effects of different IF regimens on cardiovascular events and metabolic syndrome remission. Additional studies are needed to clarify the interplay between physical activity and diet quality, and the potential impact on fertility, reproductive health, and others.

## Figures and Tables

**Table 3:** Effect of Intermittent fasting on metabolic, physical, and hormonal markers in premenopausal versus post-menopausal females.

Parameter	Premenopausal Women	Postmenopausal Women	References
Body Weight Change	−3.3 ± 0.4% (8-week TRF) −4.6 ± 3.2% (12-week ADF)	−3.3 ± 0.5% (8-week TRF) −6.5 ± 3.2% (12-week ADF)	[31,36]
Fat Mass	Decreased similarly in both groups (main effect of time, *p* < 0.05)	Decreased similarly in both groups (main effect of time, *p* < 0.05)	[31,36]
Lean Mass	Decreased similarly in both groups (main effect of time, *p* < 0.05)	Decreased similarly in both groups (main effect of time, *p* < 0.05)	[31,36]
Visceral Fat	No significant change (8-week TRF)	No significant change (8-week TRF)	[36]
Fasting Insulin	Decreased similarly in both groups (main effect of time, *p* < 0.05)	Decreased similarly in both groups (main effect of time, *p* < 0.05)	[31,36]
Insulin Resistance (HOMA-IR)	Decreased similarly in both groups (main effect of time, *p* < 0.05)	Decreased similarly in both groups (main effect of time, *p* < 0.05)	[31,36]
Fasting Glucose	No significant change (8-week TRF)	No significant change (8-week TRF) Elevated at baseline (~20% higher than premenopausal)	[36,37]
HbA1c	No significant change (8-week TRF)	No significant change (8-week TRF) Elevated at baseline (5% higher than premenopausal)	[36,38]
LDL Cholesterol	No significant change (8-week TRF) Minimal decrease (12-week ADF)	No significant change (8-week TRF) Greater decrease vs. premenopausal (12-week ADF, *p* = 0.01) Elevated at baseline	[31,36,39]
Blood Pressure	Decreased similarly in both groups (main effect of time, *p* < 0.05)	Decreased similarly in both groups (main effect of time, *p* < 0.05)	[31,36]
Testosterone/Free Androgen Index	Decreased with IF, especially with early time-restricted eating	Not measured in comparative studies	[26]
Adherence to IF Protocol	6.2 ± 0.1 days/week (8-week TRF)	6.2 ± 0.2 days/week (8-week TRF)	[36]
Postprandial Glucose Response	Lower postprandial glucose responses	42% higher postprandial glucose 4% higher peak glucose (age-matched)	[38]
Postprandial Insulin Response	Lower postprandial insulin responses	4% higher postprandial insulin	[38]

## Data Availability

All data presented in this study are available in PubMed in the public domain.

## Supplementary Materials

The following supporting information can be downloaded at: https://www.mdpi.com/article/10.3390/nu18091344/s1, Table S1: Full Database Search Strings. Table S2: Inclusion and Exclusion Criteria. Table S3: Study Characteristics of All Included References.

## Abbreviations

The following abbreviations are used in this manuscript:

HIIT	High-Intensity Interval Training
MADF	Modified Alternate Day Fasting
TRE	Time Restricted Eating
ADF	Alternate Day Fasting
IF	Intermittent Fasting
DASH	Dietary Approaches to Stop Hypertension
IHD	Ischemic Heart Disease
HDL	High-Density Lipoprotein
