# Supplementary material for: Sexual Dimorphism and Menopausal Transition: A Narrative Review of the Metabolic and Physical Effects of Intermittent Fasting
Source: Nutrients. 2026 Apr 24;18(9):1344. doi: 10.3390/nu18091344 (PMC13165054; doi:10.3390/nu18091344)
Supplement: Supplementary file 1 [file nutrients-18-01344-s001.zip › nutrients-4127948-supplementary.pdf]

SUPPLEMENTARY TABLE S1: Full Database Search Strings

Search date: December 2025.

Search logic: [Intermittent Fasting Domain] AND ([Metabolic/Cardiometabolic Domain] OR [Sex-Specific/Menopause Domain])

PubMed/MEDLINE

```
("Intermittent Fasting"[MeSH] OR "intermittent fasting"[tiab] OR "time-restricted eating"[tiab] OR "time-restricted feeding"[tiab] OR "alternate day fasting"[tiab] OR "alternate-day fasting"[tiab] OR "modified alternate day fasting"[tiab] OR "5:2 diet"[tiab] OR "16:8"[tiab] OR "periodic fasting"[tiab])
AND
(
  ("Metabolic Syndrome"[MeSH] OR "Insulin Resistance"[MeSH] OR "Obesity"[MeSH] OR "Cardiovascular Diseases"[MeSH] OR "weight loss"[tiab] OR "glycemic control"[tiab] OR "insulin sensitivity"[tiab] OR "lipid profile"[tiab] OR "blood pressure"[tiab] OR "inflammation"[tiab] OR "cardiometabolic"[tiab] OR "body composition"[tiab])
  OR
  ("Sex Characteristics"[MeSH] OR "Gonadal Hormones"[MeSH] OR "Testosterone"[MeSH] OR "Estrogens"[MeSH] OR "Kisspeptins"[MeSH] OR "Menopause"[MeSH] OR "Postmenopause"[MeSH] OR "Perimenopause"[MeSH] OR "sex differences"[tiab] OR "sexual dimorphism"[tiab] OR "menopausal transition"[tiab] OR "postmenopausal"[tiab] OR "reproductive hormones"[tiab] OR "PCOS"[tiab] OR "polycystic ovary syndrome"[tiab])
)
```

SUPPLEMENTARY TABLE S2: Inclusion and Exclusion Criteria:

Inclusion Criteria	Exclusion Criteria
Studies evaluating any form of IF (TRE, ADF, MADF, 5:2) in humans or animals	Studies not evaluating IF or a recognized fasting variant as the primary intervention
Outcomes related to body weight, insulin sensitivity, glycemic control, lipid metabolism, blood pressure, inflammatory markers, reproductive hormones, or menopausal/metabolic status	Studies with no metabolic, hormonal, or cardiometabolic outcome reported
Articles between 12/ 2015- 12 2025	Articles before 2015
Randomized controlled trials, non-inferiority trials, controlled clinical studies	Conference abstracts, editorials, letters, and opinion pieces without original data
Systematic reviews and meta-analyses of IF interventions	Studies exclusively in pediatric populations (age < 18 years)
Narrative and critical reviews providing mechanistic or clinical synthesis	Studies evaluating religious fasting without metabolic outcome data
Observational cohort studies reporting sex-specific or menopause-specific metabolic outcomes	Duplicate publications (same dataset reported in multiple papers)
Animal studies providing mechanistic evidence for sex-specific neuroendocrine responses	Non-English language publications
Articles published in English	Studies with no accessible full text

SUPPLEMENTARY TABLE S3: Study Characteristics of All Included References

Color coding: Green = EPI/Background; Blue = Mechanistic/Animal; Orange = Sex-Specific; Purple = Menopause-Specific; Yellow = Core IF Clinical Evidence.

Green rows = retrieved via database search (30 references, 69.8%). Yellow rows = identified via manual screening of reference lists (13 references, 30.2%). All manually added references are background/contextual papers without IF-specific search terms, consistent with standard practice for structured narrative reviews.

#	First Author (Year)	Journal	Design	Population/N	IF Protocol	Duration	Sex	Menopause	Role in Review
1	Murphy et al. (2021)	NCHS Data Brief	EPI	US general population; national	N/A	N/A	Both	No	Background – mortality burden
2	Naghavi Et al. (2023)	Lancet	EPI	204 countries; 660 subnational locations	N/A	N/A	Both	No	Background – global CVD burden
3	Magnussen et al. (2023)	NEJM	EPI/Cohort	~1.5M adults; global consortium	N/A	N/A	Both	No	Background – modifiable CVD risk factors
4	Chiavaroli et al. (2019)	Nutrients	SR (Umbrella)	Adults; multiple RCTs and cohorts	DASH diet	Variable	Both	No	Background – dietary patterns comparison
5	Micha et al. (2017)	JAMA	Cross-sectional	US adults; national dietary data	N/A	N/A	Both	No	Background – diet and CVD mortality
6	GBD Nordic/Baltic (2026)	Lancet Reg Health Eur	EPI	Nordic/Baltic countries	N/A	N/A	Both	No	Background – dietary burden
7	Estruch et al. (2018)	NEJM	RCT	7,447 adults at high CVD risk	Mediterranean diet + EVOO or nuts	~5 years	Both	No	Background – diet and cardiovascular disease
8	Trepanowski & Bloomer (2010)	Nutr J	NR	General population; religious fasting	Religious fasting (Ramadan, etc.)	Variable	Both	No	Background – historical/cultural/religious IF context
9	Iwayama et al. (2020)	NMR Biomed	MECH	Healthy adults; small N	Overnight fast	Single overnight	Both	No	Mechanistic – glycogen depletion physiology
10	De Cabo & Mattson (2019)	NEJM	NR	General; animal and human data	Various IF protocols	Variable	Both	No	Background/Introduction – foundational IF review
11	Fazeli & Steinhauser (2025)	Endocr Rev	CR	General; human and animal data	Various IF protocols	Variable	Both	No	Background/Discussion – critical IF appraisal
12	Varady et al. (2021)	Annu Rev Nutr	NR	Adults; human trials	ADF, TRE, 5:2	Variable	Both	No	Background – cardiometabolic IF effects
13	Navarro (2020)	Nat Rev Endocrinol	NR	General; mechanistic	N/A	N/A	Both	Partial	Mechanistic – neuroendocrine/reproductive axis
14	Mansano et al. (2023)	Endocrinology	ANIM	Female mice	Acute fasting	Acute	Female	No	Mechanistic – sex-specific kisspeptin/GABA signaling
15	Iwasa et al. (2018)	J Clin Med	NR (MECH)	General; human and animal	Low energy availability	Variable	Female	No	Mechanistic – reproductive neuroendocrine
16	Goldberg et al. (1997)	NEJM	NR/Clinical	Adults; starvation studies	Starvation/prolonged fasting	Variable	Both	No	Mechanistic – neuroendocrine starvation response
17	Patterson & Sears (2017)	Annu Rev Nutr	NR	Adults; human trials	Various IF protocols	Variable	Both	No	Background – general IF metabolic effects
18	Semnani-Azad et al. (2025)	BMJ	SR + NMA	Adults; 99 RCTs; N>6,000	ADF, TRE, 5:2, MADF	≥12 weeks	Both	No	Core evidence – IF cardiometabolic effects (highest quality)
19	Jamshed et al. (2022)	JAMA Intern Med	RCT	90 adults with obesity; mixed sex	Early TRE (8-hour window)	14 weeks	Both	No	Core evidence – TRE clinical trial

20	Guo et al. (2024)	JAMA Netw Open	RCT	405 adults with T2D	5:2 IF meal replacement	12 months	Both	No	Core evidence – IF vs pharmacotherapy in T2D
21	Lu et al. (2025)	J Health Popul Nutr	SR + MA (GRADE)	Adults with metabolic syndrome	Various IF protocols	Variable	Both	No	Core evidence – IF in metabolic syndrome (GRADE)
22	Wang et al. (2020)	Nutrition	SR + MA	Adults; 18 RCTs	Various IF protocols	Variable	Both	No	Core evidence – IF and inflammation
23	Abdollahpour et al. (2025)	Sci Rep	RCT	Adults with overweight/obesity	IF vs calorie restriction	12 weeks	Both	No	Core evidence – IF vs CER cardiovascular
24	Bosch de Basea et al. (2024)	Nutrients	ANIM	Diet-induced obese rats; male and female	IF vs caloric restriction	Variable	Both	No	Mechanistic – sex-specific IF metabolic response
25	Kumar & Kaur (2013)	PLoS One	ANIM	Young rats; male and female	IF (every other day)	3 months	Both	No	Mechanistic – sex-specific reproductive HPG effects
26	Cienfuegos et al. (2022)	Nutrients	NR (Human Trials)	Males and females; multiple human trials	Various IF protocols	Variable	Both	Partial	Core – sex-specific reproductive hormones (human)
27	Tinsley et al. (2019)	Am J Clin Nutr	RCT	40 active females	TRF (16:8) + resistance training	8 weeks	Female	No	Core – female IF + exercise body composition
28	Martínez-Rodríguez et al. (2021)	Int J Environ Res Public Health	RCT	Active women	IF + HIIT	Variable	Female	No	Core – female IF + HIIT body composition
29	Moro et al. (2016)	J Transl Med	RCT	34 resistance-trained males	TRF 16:8	8 weeks	Male	No	Core – male IF + resistance training
30	Li et al. (2021)	J Transl Med	Clinical Trial	33 women with anovulatory PCOS	8-hour TRF	5 weeks	Female	No	Core – female reproductive endocrine (PCOS)
31	Lin et al. (2021)	Nutr Metab Cardiovasc Dis	RCT (secondary analysis)	80 pre- and postmenopausal women	ADF (12 weeks)	12 weeks	Female	Yes	Core – menopause comparison (ADF)
32	Khalafi et al. (2025)	Nutrients	SR + NMA	Adults; multiple RCTs	Various IF protocols	Variable	Both	No	Core evidence – IF and inflammation (NMA)
33	Garg et al. (2025)	J Midlife Health	Review	Menopausal women	Various IF protocols	Variable	Female	Yes	Core – menopause-specific IF review
34	Valenzano et al. (2025)	Nutrients	Pilot Study	Postmenopausal women; small N	IF (protocol NR)	Variable	Female	Yes	Core – postmenopausal IF (pilot)
35	Jóźwiak et al. (2024)	J Transl Med	RCT	Menopausal women	TRE + exercise vs exercise alone	Variable	Female	Yes	Core – menopause IF + exercise RCT
36	Cienfuegos et al. (2021)	Exp Gerontol	Clinical Trial	35 pre- and postmenopausal women	TRF (8-week)	8 weeks	Female	Yes	Core – menopause comparison (TRF)
37	Donado-Pestana et al. (2025)	J Nutr Biochem	OBS	Postmenopausal women	N/A (observational)	N/A	Female	Yes	Contextual – postmenopausal metabolic baseline
38	Bermingham et al. (2022)	EBioMedicine	OBS Cohort	Pre- and postmenopausal women; ZOE PREDICT	N/A (observational)	N/A	Female	Yes	Contextual – menopause postprandial metabolism
39	Taleb-Belkadi et al. (2016)	Gynecol Endocrinol	OBS	Peri- and postmenopausal women	N/A (observational)	N/A	Female	Yes	Contextual – menopause lipid/inflammatory baseline

RCT=Randomized Controlled Trial; SR/MA=Systematic Review/Meta-Analysis; NR=Narrative Review; CR=Critical Review; OBS=Observational; MECH=Mechanistic; ANIM=Animal Study; GDL=Clinical Guideline; EPI=Epidemiological.
